# Data report on older adults from China's five national physical fitness monitoring: evidence-based psychological characteristics of ageing

**DOI:** 10.3389/fspor.2026.1691379

**Published:** 2026-02-04

**Authors:** Jiangping Fu, Yi Yu

**Affiliations:** 1 School of General Education, Nantong Institute of Technology, Nantong, Jiangsu, China; 2 School of General Education, Wuxi Normal College, Wuxi, Jiangsu, China

**Keywords:** fuzzy-set qualitative comparative analysis, motivation, national physical fitness monitoring, older adult, physical literacy, self-determination

## Abstract

**Background:**

The results of older adult data from China's five National Physical Fitness Monitoring Reports are summarized. This study examines the preliminary analysis of changes in the physical fitness of older adults in China between 2000 and 2020. The objective is to conduct evidence-based research on the psychological characteristics/health characteristics of ageing in China.

**Methods:**

Data from older adults in China's five National Physical Fitness Monitoring Reports were collected, organized, and summarized. The data for older adults includes the composite index, attainment rate, test indicators, overweight indicators, and obesity indicators. Fuzzy qualitative comparative analysis (fsQCA) was used to calculate the data for older adults. The compliance rate was set as the outcome variable (dependent variable). Composite index, attainment rate, test indicators, overweight indicators, and obesity indicators were used as conditional variables (independent variables). A comparative analysis was conducted on the characteristics of physical fitness changes in older adults (high-frequency elements and configuration).

**Results:**

In this study, the consistency threshold is set to 0.8, the PRI threshold is set to 0.75, and the frequency threshold is set to 1. The four paths (1 mixed older adult, 1 male older adult, and 2 female older adults) detected have good consistency, coverage, and robustness.

**Conclusion:**

The older adult's attainment rate is highly correlated with test indicators, overweight indicators, and obesity indicators, which is consistent with the facts. The data exposed the psychological characteristics (cognitive ability and self-determination) of older adults.

## 1 Introduction

2030 Sustainable Development Goal 3 (SDG3), sets out to ensure good health and well-being for all ages, including older adults (OA) (1). Sedentary lifestyles and lack of physical activity (PA) are obstacles to achieving SDG 3 in OA (2). The interrelationship between PA, physical fitness (PF) and health has become a hot topic in SDG 3 (3), especially among OA (4). Global ageing is expected to reach 20% (2.1 billion people) by 2050 (5), including China (5, 6). Aging brings a series of challenges to public health/services and the economy, such as population migration and national physical fitness monitoring (NPFM) (7, 8). China has adopted a systematic administrative approach to address SDG 3, such as the 2030 Healthy China Initiative (direct approach) (6) and NPFM (indirect approach) (2). The relationship between PF and good health and well-being in OA is neither unifactorial nor linear in structure (5, 8) From a complex theory perspective (9), the PF of OA is not homogeneous (10), and may be the result of multiple factors such as PA (11), physical literacy [PL (12)], and health literacy (13).

Given these age-related declines, identifying effective strategies to preserve autonomy and cognitive vitality in aging populations has become a public health priority. Therefore, our study proposes H0, which discusses the results/structure of NPFM data for OA under the premise of complex theory/PL, in order to indirectly argue the practical effectiveness of SDG3 public health/services.

Since 2000, the Chinese government has organized five NPFM (once every five years), each of which included OA (2). After each NPFM is completed, the Chinese government organizes the release of bulletins and reports to announce the results of NPFM (2). Mild cognitive impairment and cognitive decline based on the population environment are a significant age-attributable problem in OA (14). Scholars have conducted extensive research on the effects of physical exercise/activity on cognitive impairment in OA (15), such as the improvement/enhancement of cognition through different types of exercise (16, 17) and the mediation of sarcopenic obesity (18). Although physical exercise/activity can improve quality of life, from the perspective of expectation-value theory (19), the benefits for OA are not significant due to social and cultural influences. The bulletins/reports on NPFM contain the main results of the data, but they cannot guide OA in further/initial exercise interventions (2).

However, few studies have simultaneously examined the effects of remote physical training, such as NPFM, on physical, cognitive, and psychological domains, or included both active and passive control conditions to isolate the specific effects of physical exercise. Our research proposes H1, which interprets the results of NPFM data for OA based on the expectation-value theory, thereby indirectly improving OA cognitive abilities to engage in PA/PL, serving as a useful supplement to NPFM data.

Cross-cultural psychologists believe that OA' PA is the result of the cultural background of individuals or groups (20). However, NPFM as an ongoing cross-cultural encounter can bring about cultural adaptation (challenges and changes) in OA' PA (21). In an ecological context (20), during the process of cultural adaptation, individual OA must deal with/adapt to pressure sources (22, 23), triggering intrinsic motivation in self-determination theory (24). The results of NPFM data for OA (cultural background) may represent an adaptation/challenge and change individual OA and population OA. To clarify further, the results of NPFM data for OA may be a pressure source for each OA (25).

These barriers to NPFM emphasized the need for innovative approaches and gaps in delivering accessible PA interventions using digital technologies for OA, such as fuzzy qualitative comparative analysis (fsQCA) (25). Based on this, our study proposes H2, which interprets the results of NPFM data for OA based on self-determination theory, indirectly exploring how OA adapts to/interprets pressure sources (pathways), as another useful supplement to NPFM data.

Based on the above considerations, the research objectives of this study are to use the data from five NPFM in China, to perform secondary calculations, to analyze the characteristics of PF changes of Chinese OA during the period of 2000–2020, to evidence the psychological characteristics/health characteristics of aging in Chinese OA, and to complete the three proposed hypotheses (H0, H1 and H2).

## 2 Methods

### 2.1 Research design

This study conducted secondary data analysis of China's five NPFM reports for OA (2000–2020) using fsQCA. Considering the rigor of academic research, it is hereby affirmed that, according to the terms of the World Health Organization's Medical Research, cross-sectional observations (descriptive statistics), which are based on publicly available data, do not require ethical approval, and therefore, no medical ethical approvals (or numbers) were provided for this study. It is collected, organized, and summarized data from OA in China's five NPFM reports (26–29). Data for OA included composite index (CI), attainment rate (AR), test indicators (TI), overweight indicators (OvI), and obesity indicators (ObI) (30).

FsQCA was used to calculate the data for OA (31). The fsQCA 4.1 version was selected as the data calculation tool.

The AR was set as the outcome variable (dependent variable). CI, TI, OvI, and ObI were used as conditional variables (independent variables). A comparative analysis of the overall causal conditions (high-frequency factors and configurations) of independent and dependent variables was conducted as a characteristic of PF changes in OA.

Data configuration characteristics are classified according to gender into mixed OA, male OA, and female OA.

The classification of high-frequency data elements is based on three categories of configuration characteristics, combined with conditional variables, while also considering secondary sub-indicators of TI [physical indicators (PI), functional indicators (FuI), and fitness indicators (FiI)] for classification.

The high-frequency elements of the mixed OA include the comprehensive index (CI), compliance rate, TI, PI, FuI, FiI, OvI, and ObI.

The high-frequency factors for male OA include the CI, target achievement rate, male test indicators (MTI), male physical indicators (MPI), male function indicators (MFuI), male fitness indicators (MFiI), OvI, and ObI.

The high-frequency factors for female OA include the CI, target achievement rate, female test indicators (FTI), female physical indicators (FPI), female function indicators (FFuI), female fitness indicators (FFiI), OvI, and ObI.

### 2.2 Data sources/materials

The data for this study are derived from the results of China's five NPFM (2000, 2005, 2010, 2015, and 2020) (26, 32–35).

The Chinese government uses its administrative power to promote NPFM activities. The first NPFM began in 2000 and has been conducted every five years since then. Five rounds have been completed so far, with the sixth round scheduled to begin in 2025 (no data has been released).

The General Administration of Sport of China, as the functional department responsible for NPFM of the population, is specifically responsible for organizing and implementing the work and publishing the data results. Each monitoring period lasts three years, with testing conducted in the first year, data calculation in the second year, and publication of data results in the third year. The government issues bulletins and reports based on the results of each data collection.

### 2.3 Sample/population characteristics

The government's announcement was published in the form of key results, including basic information (demographic variables), CI, compliance rates, TI, and basic analysis. The government's report publicly disclosed the entire testing process (planning, organization, and implementation) as well as the data results (data broken down by age group). OA (aged 60–69) was included in all five NPFM. OvI and ObI are mentioned every time. The five NPFM data included a total of 169,894 people in OA, including 51,365 in 2000, 27,125 in 2005, 25,712 in 2010, 25,719 in 2015, and 39,973 in 2020.

In order to align with international standards, the Chinese government has developed its own testing criteria and standards based on relevant international standards. The CI uses data from 2000 as the benchmark, and counts from 100. Each subsequent test converts the data to a base value for comparison purposes. AR is a percentage value that expresses the ratio of TI to test standards for each age group, such as OA. TI are first divided into PI, FuI, and FiI; then, they are set as different tertiary indicators according to age group and gender. Body mass index (BMI) was used as an inclusion criterion to differentiate between OvI (≥24.0 kg/m^2^) and ObI (≥28.0 kg/m^2^).The TI for OA contains more detailed information (see Supplementary Table 1A).

### 2.4 Data processing/cleaning

The calculation of NPFM data is carried out by the NPFM Research Centre, which is a national research institution.

The criteria for including research data in our study include: (1) Government bulletins and reports from the General Administration of Sport of China as the primary data sources, including online (official websites) and publicly published books; (2) Data searches conducted through six databases—China National Knowledge Infrastructure, ProQuest, ERIC, ScienceDirect, Scopus, and Sport Discus—as supplementary sources; (3) Search criteria based on titles containing “NPFM/PF & OA”, with consideration given to full-text readability and downloadability, within the timeframe of 2000–2025.

It must be stated clearly and honestly that the data in government bulletins is sometimes not entirely complete. Based on our research, which is a useful supplement to government bulletins, we performed two types of data processing (cleaning). (1) Indicators (data) that cannot be directly obtained from the data in government bulletins are rated as incomplete (INC), and if unpublished data is encountered, it is marked as null frequency complete (NFC). (2) For INC data, we used averages and differences to supplement the data to meet the needs of scientific research (36).

It's about a detailed report of the procedures and details of data collection for our study. The authors formed a three-member data collection work group, including two authors and an associate professor (NPFM staff). Together, the research team developed the data categories and units of measurement for the NPFM in OA and developed an EXCEL data collection form. The first author was responsible for data collection and organization from government reports (books), and filling out the EXCEL data collection form. The second author was responsible for collecting and organizing data from government bulletins and published research papers and monographs that had been collected, and completing the EXCEL data collection form. Together, the two authors merged the EXCEL data collection form, mainly by effectively adding the second author's EXCEL data collection form to the missing items of the first author's EXCEL data collection form. Finally, the merged EXCEL data collection form was forwarded to the third data collection expert for checking and validation to determine the validity and scientific accuracy of the data.

### 2.5 Data analysis/fsQCA procedure

Comparing and analyzing the national PF data results of OA based on overall causal conditions is very complex (1 independent variable, 4 independent variables). For the analysis of overall causal conditions in complex theories, fsQCA can establish better causal configuration paths, by combining necessary conditions and configurations with high-frequency elements (37). It's about the special description of the high-frequency elements and the criteria for their inclusion. High-frequency elements are indicators with high attributes in the process of data calibration, and the criteria for their inclusion are indicators with a + mark in the intermediate solution of fsQCA.

Our research focuses on the causal structure between NPFM data for OA.

Compared with traditional analytical methods (vs. regression/correlation), fsQCA focuses on complex and unsymmetrical relationships (between results and their antecedents) (37). This is the first reason why we chose fsQCA for our research. Our study used five sets of NPFM data for OA as samples, with the year as the classification standard, for a total of five cases. The small sample size of five cases is very suitable for using fsQCA (38, 39). This is the second reason why we chose fsQCA for our research. Whether based on expectation-value theory or self-determination theory, the relationship between gender attributes (male and female OA) is usually complex, and sudden changes (differences in TI) can lead to different results. Causal (gender) asymmetry is an interesting phenomenon in the causal complexity of PF data for OA. The Boolean logic (yes or no) of fsQCA can fully explain that the causes leading to the existence of a result may be very different from those leading to the absence of a result (39). FsQCA can determine how antecedents combine with gender attributes to interpret the structure of PF data for OA (37–40). This is the third reason why we chose fsQCA for our research.

In order to capture the CI, TI, OvI, and ObI (4 independent variables) sufficient to produce results (“AR”, dependent variable), the fsQCA calibration data is first performed. Then, calculate the configuration of the five case data sets (without dividing into age groups, male OA, and female OA). The three main steps of fsQCA analysis are (1) data calibration (converting variables into fuzzy sets), (2) simplifying multiple solutions (necessity analysis of individual conditions), and (3) configuration analysis and interpretation of results (truth tables) (37–40).

## 3 Results

### 3.1 Sample characteristics

Since the first NPFM began in 2000, the Chinese government has completed five NPFMs using systematic administrative measures. The five NPFM data included a total of 169,894 people in OA, including 51,365 in 2000, 27,125 in 2005, 25,712 in 2010, 25,719 in 2015, and 39,973 in 2020.

#### 3.1.1 Raw data

Each government bulletin includes NPFM data for OA (see Supplementary Table S1). The data for OA in the NPFM includes the CI three times (not published in 2005 and 2020), AR, TI, OvI, and ObI five times each. See Supplementary Table 1B. However, not all tertiary indicators are disclosed. See Supplementary Table S1C. Statistics show that the tertiary indicators of the NPFM for OA changed during the five tests. See Supplementary Table S1.

#### 3.1.2 Variables

According to the research design, statistical analysis and calculations were performed on the stratified/grouped variables. The outcome variable (achievement rate) data from five NPFMs were obtained directly and are complete.

CI (conditional variable), 2005 value 99.39 [(2000 + 2010)/2], 2020 value 99.22 (2015 + (2015–2010). The data from five NPFM on OvI and ObI are directly obtained and complete. The TI values are calculated using data on PI, FuI, and FiI, using the formula [(PI + FuI + FiI) * 1/3]. PI includes BMI, as this data is relatively complete (2010, 2015, and 2020 data are not directly available, but can be calculated using the formula height/weight2. FuI include lung capacity, as the data is readily available and complete.

FiI included the one-leg stand with closed eyes, because only data for 2000 is missing, and comparisons of Chinese NPFM data are based on 2000. Considering scientific (minor impact), one-leg stand with closed eyes was selected; the data calculation formula is [2005-(2010–2005)].

It should be noted that the TI, PI, FuI, and FiI for the mixed OA were calculated using the average values of male and female data. The reason for this is that the bulletin did not publish specific values for the male and female OA, and according to the design principles of NPFM (equal numbers of men and women), the average value calculation method was used.

### 3.2 Data calibration

After the raw data for the condition variables and outcome variables have been determined, perform fsQCA data calibration calculations. Our study used the mean value to determine the membership of each case.

The calculation of set membership was performed using Jamovi 2.6.26 as an auxiliary calculation tool, using the direct standard method, incorporating calibration standards of 0.95 (complete membership), 0.50 (intersection point), and 0.05 (complete non-membership) (31).

Supplementary Table S2A shows the calibration information for the condition variables and outcome variables for each group in this study.

### 3.3 Necessity analysis of individual conditions

After anchor calibration, the dataset obtained a non-high set, and then a necessity analysis of individual conditions was performed. In fsQCA, when a result (high set or non-high set) occurs, a certain condition variable (high or non-high) always exists, so that condition variable is a necessary condition for the result variable (31). Consistency is an important criterion for measuring necessity (31). Scholars generally agree that when the consistency level is greater than 0.9, the condition can be considered a necessary condition for the result (31, 37–40).

Supplementary Table S2B shows the results of the necessary condition test for the AR (result variable) of different OA groups analyzed using fsQCA software. As shown in Supplementary Table S2B, among the public indicators, the consistency level of ObI (conditional variable) is 0.95 (>0.9). Therefore, ObI is a necessary condition in the conditional variables of the mixed OA, male OA, and female OA, and it should be placed first in the preliminary settings when conducting further configurational analysis (31).

As shown in Supplementary Table S2B, the consistency levels of all condition variables in the mixed OA and male OA are less than 0.9. Based on the common indicators, there is only one necessary condition (ObI) in the condition variables of both the mixed OA and male OA.

As shown in Supplementary Table S2B, in the female OA, the consistency level of ObI (conditional variable) was 0.95 (>0.9), and the consistency level of the female TI was 0.93 (>0.9); Combining the common indicators in the female OA, ObI and the female TI are both necessary conditions in the conditional variables, and when conducting further configurational analysis, they should be pre-set (placed in the first and second positions) (31, 37–40).

### 3.4 Configuration analysis

In the calculation process of the truth table algorithm, our study set the consistency threshold to 0.8, the PRI threshold to 0.75, and the frequency threshold to 1 (31). In fsQCA, there are three solutions/configurations (i.e., complex solution, intermediate solution, and simple solution) in the sufficiency analysis of configuration conditions. Furthermore, intermediate solutions are preferable to complex solution and simplified solution (31, 37–40). Our study mainly reports intermediate solutions, supplemented by simplified solutions, to determine the stratification configuration of OA (mixed OA, male OA, and female OA).

The analysis results show that the mixed OA path only analyzed one configuration OA, but the consistency level 0.88 was higher than the minimum acceptable standard 0.75, and the overall scheme coverage was 0.30, which is a good result. See Supplementary Table S2C.

The analysis results show that only one configuration was analyzed for the male OA, but the consistency level 0.88 was higher than the minimum acceptable standard 0.75, and the overall scheme coverage was 0.28, indicating good data results. See Supplementary Table S2C.

The analysis results show that two configurations were identified in the female OA path analysis. Both the individual configurations (1 configuration 1, 0.87 configuration 2) and the overall solution (0.93) had a consistency level higher than the minimum acceptable standard of 0.75. The overall scheme coverage was 0.79, and the data results were good. See Supplementary Table S2C.

### 3.5 Robustness testing

Post-hoc robustness tests are an effective way to avoid randomness and sensitivity in results (31). Our study used a robust test by adjusting the consistency threshold, specifically, by lowering the consistency threshold from 0.80 to 0.75 (31). The adjusted results are consistent with the original results in terms of the state of the set relationship and the fitting parameters. Therefore, the researchers believe that the data analysis results of this study are robust.

## 4 Discussion

For the secondary calculation of the five NPFM data sets for OA, the fsQCA method was used to obtain one mixed OA configuration (∼ObI*CI*TI*∼PI*FuI*FiI*∼OvI), one male OA configuration (∼OBI*CI*∼MTI* ∼MPI*MFUI*MFII*∼OvI), and female OA 2 configuration (∼FTI*∼OBI*CR*∼FPI*∼FFUI*FFII*∼OvI, FTI*OBI*∼CR*∼FPI*FFUI*∼FFII*OvI).

After calculating the necessity of individual conditions, we observed that ObI was a necessary condition for all OAs (mixed OA, male OA, and female OA), which indicates that ObI is a necessary condition for the PF of OA. In other words, in order to improve the PF of OA, the impact of ObI must be given priority consideration.

This result is consistent with logic, as a low ObI can predict high PF.

After calculating the necessity of individual conditions, we also observed that there is another necessary condition for the PF of female OA, which is high FPF. This shows that high FPF is the preferred condition for improving the PF of female OA. In other words, the improvement of the PF of female OA is achieved by improving their PF.

Of course, both necessary conditions must be considered simultaneously. The PF of female OA can be improved by enhancing their PI and reducing their ObI.

This is an important supplement to the interpretation (significance and value) of NPFM data.

### 4.1 Complex theories and PL

From a complex theory perspective (41), the interpretation of the results of NPFM for OA is complex and may have non-linear characteristics. Through fsQCA calculations, this study observed that the mixed OA has one configuration, consisting of all conditioning variables. This configuration shows a nonlinear logistic relationship between the outcome variable (AR) and the conditional variables (CI, TI, PI, FuI, FiI, OvI, and ObI) for all OA. In order to better name the configurations, our study used the perspective of PL theory (concept). PL concepts (indicators) are important indicators for China's goal of becoming a sports powerhouse by 2030. The International PL Alliance (IPLA) defines PL as the motivation, confidence, PA, knowledge, and understanding necessary for lifelong participation in PA, as well as the intrinsic drive to take responsibility for it. OA PL has attracted the attention of many scholars. Some believe that PL can improve/alleviate chronic diseases in OA, thereby improving their PF.

Our study linked the NPFM indicators for OA with PL, recognizing that the achievement rate represents PL, the CI represents motivation, the TI (including PI, FuI, and FiI) represents PA, and the OvI/ObI represent knowledge and understanding. See Supplementary Table S3A. Our study associated the configuration of the mixed OA in fsQCA with PL. The PL path (configuration) of the mixed OA had 7 strong/weak combinations (+4, ∼3), and strong PL (+4) was observed to be greater than weak PL (∼3). OA PL pathways (configuration) exhibit characteristics of strong motivation (CI), strong confidence (test index), strong PA (function), and strong PA (fitness), as well as characteristics of weak PA (physical), weak knowledge and understanding (overweight index and obesity index).

This may be a useful addition to the interpretation (value and significance) of NPFM data for OA.

### 4.2 Expectancy-value theory

The results of NPFM for OA showed different characteristics depending on gender. During the test, OA was divided into male and female. The outcome variables (achievement rate/PL) for the male OA and female OA may have different characteristics. This study introduced the expectation-value theory (42–44) observed the structural characteristics of the PL pathways (outcome variables and condition variables) of the male OA and female OA. It is important to note that there are two necessary conditions for female OA: high test scores and low ObI. Therefore, we included configuration 2 (FTI* ObI *∼CR*∼FPI*FFUI*∼FFII*OvI) as the observation data. See Supplementary Table S3B.

In expectation-value theory, motivation = expectation * value (43).

In our study, we used the PL path of OA to represent their motivation for PL (rate of meeting standards/high PF), strong PL (+) to represent their expectations for PF, and weak PL (∼) to represent the value of PF for OA. After calculation, it can be observed from Supplementary Table S3B that the motivation (expectancy-value theory) of the male OA and the female OA is 12, expressing that the motivation of male and female OA for PL (high standard rate/high PF) is the same, and also expressing that the pursuit of high PF/high standard rate by OA is the same. However, Supplementary Table S3B also shows that the strong PL (+3) of the male OA is lower than that (+4) of the female OA, indicating that male OA may have lower expectations for high standards/high PF than female OA.

This may be a useful third addition to the interpretation (value and significance) of NPFM data for OA.

### 4.3 Self-determination theory

The results of NPFM for male and female OA (different structures) show that, in addition to gender differences, how OA of the same gender determines their PA behavior ultimately shapes their PF results. This is the subject of further discussion in our study.

In self-determination theory, there are three common psychological needs that must be met in order for individuals to feel psychologically fulfilled (competence, autonomy, and relatedness) (44, 45). Previous studies have suggested that individuals will only develop intrinsic motivation and exhibit behavior (Regular PA or exercise) when these three needs are met (44–46).

Our study uses the outcome variable (achievement rate) to represent the PF behavior of OA, the PL path (configuration) to represent the intrinsic motivation of the PF outcomes of OA, strong PL (+) to represent competitiveness (psychological needs), the difference between strong PL and weak PL (formula, autonomy = strong PL—weak PL) to represent autonomy (psychological needs), and the percentage of the frequency of strong PL (Formula: Relatedness = Strong PL/[Strong PL + Weak PL)] to represent relatedness (psychological need).

The psychological source/self-determination characteristics of the PL path (configuration) of the male OA are competitiveness 3, autonomy −1, and 42.86% correlation with strong PL. The psychological source/self-determination characteristics of PL pathway 1 (configuration 1) in the female OA are competitiveness 2, autonomy 3, and 28.57% correlation with strong PL. The psychological source/self-determination characteristics of PL pathway 2 (configuration 2) in the female OA are competitiveness 4, autonomy 1, and 57.14% correlation with strong PL.

This (Supplementary Table S3C) may be a useful fourth addition to the interpretation (value and significance) of NPFM data for OA.

### 4.4 Advantages limitations and future direction

**Advantages:** Our research provides a detailed logical framework for interpreting NPFM data. This logical framework may be the greatest strength of our research. In addition, the results of our study provide four useful additions to the interpretation (value and significance) of NPFM data for OA.

**Limitations:** Since the main source of raw data is bulletins and reports published by government departments, some data is INC or NFC. Although we have performed logical calculations, there are limitations. All data results can only represent the results of this study.

**Future direction:** PF monitoring for OA is a dynamic process. As a health promotion, it is vital to encompass active, equitable and inclusive ageing in order to promote health (47). The Chinese government is promoting the smooth implementation of NPFM for OA through systematic administrative measures, which require continued attention. The PL indicators proposed in China's 2030 strategy to become a sporting powerhouse may guide the PF of OA towards a beneficial direction.

## Data Availability

The original contributions presented in the study are included in the article/Supplementary Material, further inquiries can be directed to the corresponding author.
