# Supplementary material for: Data report on older adults from China's five national physical fitness monitoring: evidence-based psychological characteristics of ageing
Source: Front Sports Act Living. 2026 Feb 4;8:1691379. doi: 10.3389/fspor.2026.1691379 (PMC12914949; doi:10.3389/fspor.2026.1691379)
Supplement: Supplementary file 1 [file Table1.docx]

Supplementary Material

# Supplementary Tables 1

**TABLE 1 Data for national physical fitness monitoring indicators for older adults.**

1. **Statistical Table of Test Indicators for older adults.**

| **Indicators** |  | **2000** | **2005** | **2010** | **2015** | **2020** |
| --- | --- | --- | --- | --- | --- | --- |
| Physical indicators |  |  |  |  |  |  |
| Basic indicators |  |  |  |  |  |  |
|  | height | # | # | # | # | # |
|  | weight | # | # | # | # | # |
|  | chest size | # | # | # | # | # |
|  | waist size | # | # | # | # | # |
|  | hip size | # | # | # | # | # |
|  | upper arm skinfold thickness | # | # | # | # | NFC |
|  | scapular skinfold thickness | # | # | # | # | NFC |
|  | abdominal skinfold thickness | # | # | # | # | NFC |
| Derived indicators |  |  |  |  |  |  |
|  | BMI | # | # | # | # | NFC |
|  | weight/height × 1000 | # | # | # | # | NFC |
|  | chest /height × 100 | # | # | # | # | NFC |
|  | skinfold thickness | # | # | # | # | NFC |
|  | waist / hip*100 | # | # | # | # | NFC |
|  | waist/chest*100 | # | # | # | # | NFC |
|  | hip/height*100 | # | # | # | # | NFC |
|  | fat rate | - | - | - | - | # |
| Function Indicators |  |  |  |  |  |  |
|  | pulse rate | # | # | # | # | NFC |
|  | systolic blood pressure | # | # | # | # | NFC |
|  | diastolic blood pressure | # | # | # | # | NFC |
|  | lung capacity | # | # | # | # | # |
|  | lung capacity/height | - | # | # | # | NFC |
|  | 2-minute high-knee running in place | - | - | - | - | # |
| Fitness indicators |  |  |  |  |  |  |
|  | sit-and-reach | # | # | # | # | # |
|  | grip strength | # | # | # | # | # |
|  | one-leg stand with closed eyes | # | # | # | # | # |
|  | choice reaction time | # | # | # | # | # |

**Remarks:** -: No annual tests indicator; #: Annual test indicator; NFC: Unpublished data.

1. **Statistical table of monitoring indicators and variables for older adults.**

| **Indicators** | **2000** | **2005** | **2010** | **2015** | **2020** | Variables |
| --- | --- | --- | --- | --- | --- | --- |
| Attainment rate | 100 | NFC/99.39 | 98.78 | 99 | NFC/99.22 | OV |
| Composite index | INC/576.5 | INC/733.78 | INC/713.44 | INC/799.52 | INC/704.88 | CVAG |
| Overweight indicators | 10.85 | 11.3 | 13 | 13.9 | 16.7 | CVAG |
| Obesity indicators | INC/23.05 | INC/24.95 | INC/24.4 | INC/24.605 | INC/25.03 | CVAG |
| Testing indicators | 34.59 | 35.6 | 39.8 | 41.6 | 41.7 | CVMI |
| Physical indicators | INC/2200 | INC/2165 | INC/2107.25 | INC/2365.5 | INC/2079 | CVMI |
| Function Indicators | INC/14.125 | INC/11.4 | INC/8.675 | INC/8.45 | INC/10.6 | CVMI |
| Fitness indicators | INC/574.37 | INC/895.8 | INC/847.43 | INC/992.07 | INC/820.59 | CVMI |
| Male test indicators | INC/23 | INC/25.1 | INC/24.14 | INC/24.42 | INC/24.98 | CVMG |
| Male physical indicators | INC/2700 | INC/2650 | INC/2509 | INC/2943 | INC/2426 | CVMG |
| Male function Indicators | INC/15.45 | INC/12.3 | INC/9.15 | INC/8.8 | INC/10.8 | CVMG |
| Male fitness indicators | INC/578.63 | INC/571.77 | INC/579.45 | INC/606.96 | INC/589.16 | CVMG |
| Female test indicators | INC/23.1 | INC/24.8 | INC/24.66 | INC/24.79 | INC/25.08 | CVFG |
| Female physical indicators | INC/1700 | INC/1680 | INC/1705.5 | INC/1788 | INC/1732 | CVFG |
| Female function Indicators | INC/12.8 | INC/10.5 | INC/8.2 | INC/8.1 | INC/10.4 | CVFG |
| Female fitness indicators | 100 | NFC/99.39 | 98.78 | 99 | NFC/99.22 | CVFG |

**Remarks:** OV: Outcome variables; CVAG: Conditional variables (all groups); CVMI: Conditional variables (mixed group); CVMG: Conditional variables (male group); CVFG: Conditional variables (female group).

1. **Statistical Table of Test Indicators for older adults.**

| **Indi-cators** | **2000** |  | **2005** |  | **2010** |  | **2015** |  | **2020** |  |
| --- | --- | --- | --- | --- | --- | --- | --- | --- | --- | --- |
|  | **Male** | **Female** | **Male** | **Female** | **Male** | **Female** | **Male** | **Female** | **Male** | **Female** |
| PI |  |  |  |  |  |  |  |  |  |  |
| H1 | 165.7 | 154.5 | NFC | NFC | 165.3 | 153.95 | 165.75 | 154.85 | 165.65 | 154.75 |
| W1 | 63.1 | 55.2 | NFC | NFC | 65.95 | 58.45 | 67.1 | 59.45 | 68.55 | 60.05 |
| CZ1 | NFC | NFC | NFC | NFC | 91.35 | 89.05 | 92.4 | 90.5 | NFC | NFC |
| WZ1 | 84.2 | 80.1 | 86.5 | 84.2 | 85.35 | 84 | 86.9 | 85.1 | 89.3 | 85.95 |
| HZ1 | NFC | NFC | NFC | NFC | 92.9 | 93.75 | 94.1 | 96.1 | 96.35 | 95.35 |
| UAST1 | NFC | NFC | NFC | NFC | 11.1 | 19.6 | 12.15 | 19.65 | INC | INC |
| SST1 | NFC | NFC | NFC | NFC | 16.45 | 20.55 | 17.55 | 21 | NFC | NFC |
| AST1 | NFC | NFC | NFC | NFC | 20.45 | 26.4 | 22.05 | 27.2 | INC | INC |
| BMI2 | 23 | 23.1 | 25.1 | 24.8 | INC/24.14 | INC/24.66 | INC/24.42 | INC/24.79 | INC/24.98 | INC/25.08 |
| W/H2 | INC | INC | INC | INC | INC | INC | INC | INC | INC | INC |
| C/H2 | INC | INC | INC | INC | INC | INC | INC | INC | INC | INC |
| ST2 | INC | INC | INC | INC | INC | INC | INC | INC | INC | INC |
| W/HI2 | INC | INC | INC | INC | INC | INC | INC | INC | INC | INC |
| W/C2 | INC | INC | INC | INC | INC | INC | INC | INC | INC | INC |
| H/H2 | INC | INC | INC | INC | INC | INC | INC | INC | INC | INC |
| FR2 | NFC | NFC | NFC | NFC | NFC | NFC | NFC | NFC | INC | INC |
| FI |  |  |  |  |  |  |  |  |  |  |
| PR | NFC | NFC | NFC | NFC | 75.85 | 75.6 | 77 | 76.6 | INC | INC |
| SBP | NFC | NFC | NFC | NFC | 131.1 | 129.25 | 130.2 | 128.15 | INC | INC |
| DBP | NFC | NFC | NFC | NFC | 81.4 | 79.3 | 80.6 | 77.85 | NFC | NFC |
| LC | 2700 | 1700 | 2650 | 1680 | 2509 | 1705.5 | 2943 | 1788 | 2426 | 1732 |
| LC/H | INC | INC | INC | INC | INC | INC | INC | INC | INC | INC |
| HKR | NFC | NFC | NFC | NFC | NFC | NFC | NFC | NFC | 51.25 | 53.7 |
| FIN |  |  |  |  |  |  |  |  |  |  |
| SAR | 3.2 | 5.1 | NFC | NFC | 1.15 | 7.3 | 1.8 | 7.55 | 2.05 | 7.5 |
| GR | 34.2 | 21.8 | NFC | NFC | 36 | 22.55 | 36.15 | 22.75 | 35.8 | 23.2 |
| OLS | NFC/15.45 | NFC/12.8 | 12.3 | 10.5 | 9.15 | 8.2 | 8.8 | 8.1 | 10.8 | 10.4 |
| CRT | NFC | NFC | NFC | NFC | 0.7 | 0.75 | 0.69 | 0.73 | 0.73 | 0.76 |

**Remarks:** PI: Physical indicators; H: height; W: weight; CZ: chest size; WZ: waist size; HZ: hip size; UAST: upper arm skinfold thickness; SST: scapular skinfold thickness; AST: abdominal skinfold thickness; W/H: weight/height × 1000; C/H: chest /height × 100; S/T: skinfold thickness; W/HI: waist / hip*100; W/C: waist/chest*100; H/H: hip/height*100; FR: fat rate; FI: Function Indicators; PR: pulse rate; SBP: systolic blood pressure; DBP: diastolic blood pressure; LC: lung capacity; LC/H: lung capacity/height; HKI: 2-minute high-knee running in place; FIN: Fitness indicators; SAR: sit-and-reach; GR: grip strength; OLS: one-leg stand with closed eyes; CRT: choice reaction time; ^1^: Basic indicators; ^2^: Derived indicators ; INC: incomplete data ；NFC: Unpublished data.
