# Supplementary material for: Data report on older adults from China's five national physical fitness monitoring: evidence-based psychological characteristics of ageing
Source: Front Sports Act Living. 2026 Feb 4;8:1691379. doi: 10.3389/fspor.2026.1691379 (PMC12914949; doi:10.3389/fspor.2026.1691379)
Supplement: Supplementary file 2 [file Table2.docx]

Supplementary Material

# Supplementary Table 2

**TABLE 2. FsQCA of national physical fitness monitoring data for older adults.**

1. **Calibration anchors for condition variables and result variables**.

| **Variables** | | **Target set** | | **Completely dependent** | | **Intersection point** | | **Completely independent** | |
| --- | --- | --- | --- | --- | --- | --- | --- | --- | --- |
|  | |  | | **Affiliation degree 0.95** | | **Affiliation degree 0.50** | | **Affiliation degree 0.05** | |
| Outcome variables |  | |  | |  | |  | |  |
| Attainment rate | | High Attainment rate | | 84.8 | | 86.4 | | 90.5 | |
| Conditional variables (all groups) |  | |  | |  | |  | |  |
| Composite index | | High composite index | | 98.8 | | 99.2 | | 99.9 | |
| Overweight indicators | | High overweight indicators | | 34.8 | | 39.8 | | 41.7 | |
| Obesity indicators | | High Obesity indicators | | 10.9 | | 13 | | 16.1 | |
| Conditional variables (mixed group) | |  | |  | |  | |  | |
| Testing indicators | | High testing indicators | | 707 | | 734 | | 789 | |
| Physical indicators | | High physical indicators | | 23.3 | | 24.6 | | 25 | |
| Function Indicators | | High function Indicators | | 2085 | | 2165 | | 2332 | |
| Fitness indicators | | High fitness indicators | | 8.5 | | 10.6 | | 13.6 | |
| Conditional variables (male group) | |  | |  | |  | |  | |
| Male test indicators | | High male test indicators | | 624 | | 847 | | 973 | |
| Male physical indicators | | High male physical indicators | | 23.2 | | 24.4 | | 25.1 | |
| Male function Indicators | | High male function Indicators | | 2443 | | 2650 | | 2894 | |
| Male fitness indicators | | High male fitness indicators | | 8.87 | | 10.8 | | 14.8 | |
| Conditional variables (female group) | |  | |  | |  | |  | |
| Female test indicators | | High female test indicators | | 573 | | 579 | | 603 | |
| Female physical indicators | | High female physical indicators | | 23.4 | | 24.8 | | 25 | |
| Female function Indicators | | High female function Indicators | | 1684 | | 1706 | | 1777 | |
| Female fitness indicators | | High female fitness indicators | | 8.12 | | 10.4 | | 12.3 | |

**(B) Necessary condition analysis of attainment rate (outcome variable) in older adults**.

| **Conditional variables** | | **High attainment rate** | | **Low attainment rate** | |
| --- | --- | --- | --- | --- | --- |
|  | **Code** | **Consistency** | **Coverage** | **Consistency** | **Coverage** |
| **Conditional variables (all groups)** | | | | | |
| High composite index | CI | 0.470817 | 0.504167 | 0.613169 | 0.620833 |
| Low composite index | ~CI | 0.645914 | 0.638462 | 0.510288 | 0.476923 |
| High overweight indicators | OvI | 0.832685 | 0.856000 | 0.415638 | 0.404000 |
| Low overweight indicators | ~OvI | 0.420233 | 0.432000 | 0.851852 | 0.828000 |
| High obesity indicators | ObI | 0.840467 | 0.943232 | 0.419753 | 0.445415 |
| Low obesity indicators | ~ObI | 0.505837 | 0.479705 | 0.946502 | 0.848708 |
| **Conditional variables (mixed group)** | | | | | |
| High testing indicators | TI | 0.478599 | 0.549107 | 0.62963 | 0.683036 |
| Low testing indicators | ~TI | 0.723735 | 0.673913 | 0.584362 | 0.514493 |
| High physical indicators | PI | 0.747082 | 0.678445 | 0.720165 | 0.618375 |
| Low physical indicators | ~PI | 0.579767 | 0.686636 | 0.625514 | 0.700461 |
| High function Indicators | FuI | 0.48249 | 0.548673 | 0.63786 | 0.685841 |
| Low function Indicators | ~FuI | 0.723735 | 0.678832 | 0.580247 | 0.514599 |
| High fitness indicators | FiI | 0.416342 | 0.473451 | 0.563786 | 0.606195 |
| Low fitness indicators | ~FiI | 0.653696 | 0.613139 | 0.510288 | 0.452555 |
| **Conditional variables (male group)** | | | | | |
| High male test indicators | MTI | 0.614786 | 0.593985 | 0.691358 | 0.631579 |
| Low male test indicators | ~MTI | 0.618677 | 0.679487 | 0.555556 | 0.576923 |
| High male physical indicators | MPI | 0.712062 | 0.663043 | 0.707819 | 0.623188 |
| Low male physical indicators | ~MPI | 0.595331 | 0.683036 | 0.617284 | 0.669643 |
| High male function Indicators | MFUI | 0.486381 | 0.550661 | 0.641975 | 0.687225 |
| Low male function Indicators | ~MFII | 0.653696 | 0.629214 | 0.580247 | 0.516483 |
| High male fitness indicators | MFII | 0.420233 | 0.463519 | 0.592593 | 0.618026 |
| Low male fitness indicators | ~MFII | 0.653696 | 0.629214 | 0.485597 | 0.441948 |
| **Conditional variables (female group)** | | | | | |
| High female test indicators | FTI | 0.926070 | 0.868613 | 0.567901 | 0.50365 |
| Low female test indicators | ~FTI | 0.470817 | 0.535398 | 0.851852 | 0.915929 |
| High female physical indicators | FPI | 0.754864 | 0.795082 | 0.559671 | 0.557377 |
| Low female physical indicators | ~FPI | 0.579767 | 0.582031 | 0.794239 | 0.753906 |
| High female function Indicators | FFUI | 0.852140 | 0.862205 | 0.502058 | 0.480315 |
| Low female function Indicators | ~FFUI | 0.486381 | 0.50813 | 0.855967 | 0.845528 |
| High female fitness indicators | FFII | 0.416342 | 0.504717 | 0.502058 | 0.575472 |
| Low female fitness indicators | ~FFII | 0.649805 | 0.579861 | 0.567901 | 0.479167 |

**Remarks:** *, necessary condition, consistency ≥ 0.9.

**(C) Configuration analysis of national physical fitness monitoring for older adults**.

| **(A) Mixed** |  | **(B) Male** |  | **(C) Female** |  |  |
| --- | --- | --- | --- | --- | --- | --- |
| **CV** | **CC（1）** | **CV** | **CC（1）** | **CV** | **CC（1）** | **CC（2）** |
| CI | ⊙ | CI | ⊙ | CI | ∽ | ∽ |
| TI | ⊙ | MTI | ○ | FTI | Θ | ⊕ |
| PI | ○ | MPI | ○ | FPI | ○ | ○ |
| FuI | ⊙ | MFuI | ⊙ | FFuI | ∽ | ∽ |
| FiI | ⊙ | MFiI | ⊙ | FFiI | ∽ | ∽ |
| OvI | ⊕ | OvI | ⊕ | OvI | ∽ | ∽ |
| ObI | Θ | ObI | Θ | ObI | ⊕ | Θ |
| raw coverage | 0.300412 |  | 0.288066 |  | 0.465021 | 0.374486 |
| unique coverage | 0.300412 |  | 0.288066 |  | 0.411523 | 0.320988 |
| consistency | 0.879518 |  | 0.875000 |  | 1.000000 | 0.866667 |
| solution coverage | 0.300412 |  | 0.288066 |  | 0.786008 |  |
| solution consistency | 0.879518 |  | 0.875000 |  | 0.931707 |  |

**Remarks:** CV: Conditional variables; CC: Condition configuration; ⊕= core condition present; Θ= core condition absent; ⊙ = auxiliary condition present; ○ = auxiliary condition absent; ‘~’ indicates that the condition may or may not be present.
