# Supplementary material for: Data report on older adults from China's five national physical fitness monitoring: evidence-based psychological characteristics of ageing
Source: Front Sports Act Living. 2026 Feb 4;8:1691379. doi: 10.3389/fspor.2026.1691379 (PMC12914949; doi:10.3389/fspor.2026.1691379)
Supplement: Supplementary file 3 [file Table3.docx]

Supplementary Material

# Supplementary Table 3

**TABLE 3. Analysis table of national physical fitness monitoring data for older adults**.

**(A) Physical fitness configuration path for mixed older adults.**

| **Conditional variables** |  | **Physical literacy** |  |  |
| --- | --- | --- | --- | --- |
| **Code** | **Configuration** | **Variables** | **+(High)** | **-(Low)** |
| CI | + | Motivation | 1 | 0 |
| TI | + | Confidence | 1 | 0 |
| PI | ~ | Physical ability | 0 | 1 |
| FuI | + |  | 1 | 0 |
| FiI | + |  | 1 | 0 |
| OvI | ~ | KU | 0 | 1 |
| ObI | ~ |  | 0 | 1 |
| Total | 7 | 4 | 4 | 3 |

**Remarks:** KU: Knowledge and understanding.

**(B) Physical literacy configuration pathways for older adults (expectation-value theory).**

| **CV** | **Male** | **Female** | **MPL** |  | **FPL** |  |
| --- | --- | --- | --- | --- | --- | --- |
| **Code** | **CON** | **CON (2)** | **+(High)** | **-(Low)** | **+(High)** | **-(Low)** |
| CI | + | ~ | 1 | 0 | 0 | 1 |
| MTI/FTI | ~ | + | 0 | 1 | 1 | 0 |
| MPI/FPI | ~ | ~ | 0 | 1 | 0 | 1 |
| MFuI/Fful | + | + | 1 | 0 | 1 | 0 |
| MFiI/Ffil | + | ~ | 1 | 0 | 0 | 1 |
| OvI | ~ | + | 0 | 1 | 1 | 0 |
| ObI | ~ | + | 0 | 1 | 1 | 0 |
| Total | 7 | 7 | 3 | 4 | 4 | 3 |
| Motivation |  |  | 12 |  | 12 |  |

**Remarks: CV: Conditional variables; CON: Configuration; MPL: Male physical literacy; FPL: Female physical literacy.**

**(C) Physical literacy configuration pathways for older adults (self-determination theory).**

| **CV** | **Male** | **Female** |  | **MPL** |  | **FPL1** |  | **FPL2** |  |
| --- | --- | --- | --- | --- | --- | --- | --- | --- | --- |
| **Code** | **CON** | **CON (1)** | **CON (2)** | **+(High)** | **-(Low)** | **+(High)** | **-(Low)** | **+(High)** | **-(Low)** |
| CI | + | + | ~ | 1 | 0 | 1 | 0 | 0 | 1 |
| MTI/FTI | ~ | ~ | + | 0 | 1 | 0 | 1 | 1 | 0 |
| MPI/FPI | ~ | ~ | ~ | 0 | 1 | 0 | 1 | 0 | 1 |
| MFuI/Fful | + | ~ | + | 1 | 0 | 0 | 1 | 1 | 0 |
| MFiI/Ffil | + | + | ~ | 1 | 0 | 1 | 0 | 0 | 1 |
| OvI | ~ | ~ | + | 0 | 1 | 0 | 1 | 1 | 0 |
| ObI | ~ | ~ | + | 0 | 1 | 0 | 1 | 1 | 0 |
| Total | 7 | 7 | 7 | 3 | 4 | 2 | 5 | 4 | 3 |
| COM |  |  |  | 3 |  | 2 |  | 4 |  |
| AU |  |  |  | -1 |  | -3 |  | 1 |  |
| RE |  |  |  | 0.43 | 0.57 | 0.29 | 0.71 | 0.57 | 0.423 |

**Remarks:** CV: Conditional variables; CON: Configuration; MPL: Male physical literacy; FPL: Female physical literacy; COM: Competitiveness; AU: Autonomy; RE: Relevance.
